# Nanoscale solely amorphous layer in silicon wafers induced by a newly developed diamond wheel

**DOI:** 10.1038/srep35269

**Published:** 2016-10-13

**Authors:** Zhenyu Zhang, Liangchao Guo, Junfeng Cui, Bo Wang, Renke Kang, Dongming Guo

**Affiliations:** 1 Key Laboratory for Precision and Non-Traditional Machining Technology of Ministry of Education, Dalian University of Technology, Dalian 116024, China; 2 Changzhou Institute of Dalian University of Technology, Changzhou 213164, China

## Abstract

Nanoscale solely amorphous layer is achieved in silicon (Si) wafers, using a developed diamond wheel with ceria, which is confirmed by high resolution transmission electron microscopy (HRTEM). This is different from previous reports of ultraprecision grinding, nanoindentation and nanoscratch, in which an amorphous layer at the top, followed by a crystalline damaged layer beneath. The thicknesses of amorphous layer are 43 and 48 nm at infeed rates of 8 and 15 μm/min, respectively, which is verified using HRTEM. Diamond-cubic Si-I phase is verified in Si wafers using selected area electron diffraction patterns, indicating the absence of high pressure phases. Ceria plays an important role in the diamond wheel for achieving ultrasmooth and bright surfaces using ultraprecision grinding.

Silicon (Si) wafers are widely used substrates for fabricating more than 90% of the semiconductor devices^1^. Si has dominated both the semiconductor and electronics industries for several decades^2^,^3^. Silicon wafers experience successive machining processes after slicing from an ingot, such as lapping, ultraprecision grinding, chemical mechanical polishing (CMP), etc. Ultraprecision grinding has unique advantages in efficiency and accuracy, compared to lapping and CMP. Composite damaged layer forms in Si wafers after ultraprecision grinding, consisting of an amorphous layer at the top and a crystalline damaged layer beneath^4^,^5^. The composite damaged layer is consistent with those formed in nanoindentation^6^,^7^,^8^ and nanoscratch^9^. Moreover, high pressure phases, such as Si-III and Si-XII, referring to body-centered cubic (bc8) and rhombohedral (r8) phases, respectively are reported in nanoindentation^6^,^7^ and nanoscratch^9^. Nevertheless, damage-free and surface roughness less than 1 nm are required for a Si wafer to become a qualified substrate for a high-performance device. Therefore, the composted damaged layer needs to be removed in a Si wafer, despite an amorphous layer at the top, followed by a crystalline damaged layer underneath, induced by the ultraprecision grinding^4^,^5^, nanoindentation^6^,^7^,^8^ and nanoscratch^9^.

The thinner of the composite damaged layer generated by ultraprecision grinding is, the less cost and time for the subsequent CMP are. A solely crystalline damaged layer is found in Si under nanoindentation, but its thickness is approximately 4 μm^10^. The thickness of a crystalline damaged layer is usually several times that of an amorphous layer generated under nanoindentation and nanoscartch^7^,^8^,^9^. Thus, a solely amorphous layer is expected for the best outcome by abrasive machining, whereas it has not been reported by ultraprecision grinding performed using a diamond wheel. Nonetheless, the solely amorphous layer with thickness of 10 nm is obtained by CMP, rather than ultraprecision grinding by a diamond wheel^11^. It is intriguing to develop a novel diamond wheel performing high-performance grinding, i.e. nanoscale solely amorphous layer left in Si wafers after grinding, which is beneficial for both the semiconductor and electronics industries.

In this study, two novel resin bond diamond wheels are developed with ceria (CeO_2_) and silica (SiO_2_), respectively. Nanoscale solely amorphous layer is obtained using the newly developed diamond wheel with ceria. The nanoscale solely amorphous layer is confirmed using high resolution transmission electron microscopy (HRTEM), and the fundamental mechanism of ultraprecision grinding is investigated.

## Results

**Figure 1:**
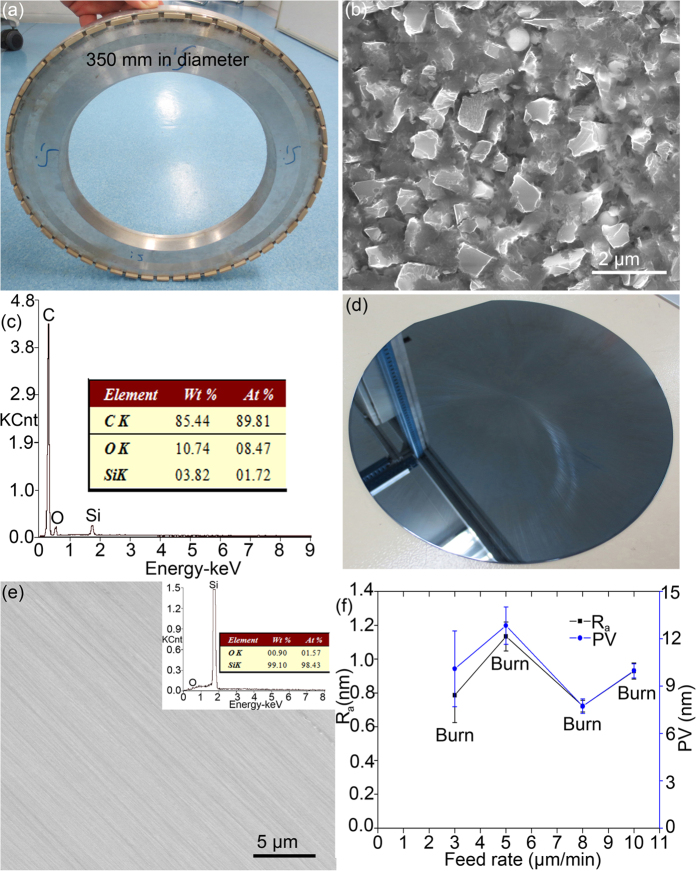
Optical (**a**) and SEM (**b**) images of Sample S1, (**c**) its EDS spectrum in (**b**), optical (**d**) and SEM (**e**) images on a ground Si wafer at a feed rate of 8 μm/min, and (**f**) surface roughness R_a_ and PV as a function of feed rate on the Si wafers ground by Sample S1. The inset in (**e**) showing its corresponding EDS spectrum.

Figure 1 shows the optical and scanning electron microscopy (SEM) images of Sample S1 (Table 1), its energy dispersive spectroscopy (EDS) spectrum, optical and SEM images on a ground Si wafer, and surface roughness R_a_ and PV as a function of feed rate on the Si wafers ground by Sample S1. Diamond grains are fixed by the resin bond (Fig. 1(b)). The weight percentage of SiO_2_ in Sample S1 was 8.2% (Table 1), which is in a good agreement with the EDS spectrum in Fig. 1(c). SiO_2_ is not obvious in the resin bond (Fig. 1(b)). The ground Si wafer in Fig. 1(d) is dark and burnt, resulting in an increase in the oxygen content on the Si surface (Fig. 1(e)). Grinding marks are present on the ground Si surface in Fig. 1(e). Burn takes place on all the Si wafers ground by Sample S1, as shown in Fig. 1(f), indicating inability of Sample S1 for grinding Si wafers.

**Figure 2:**
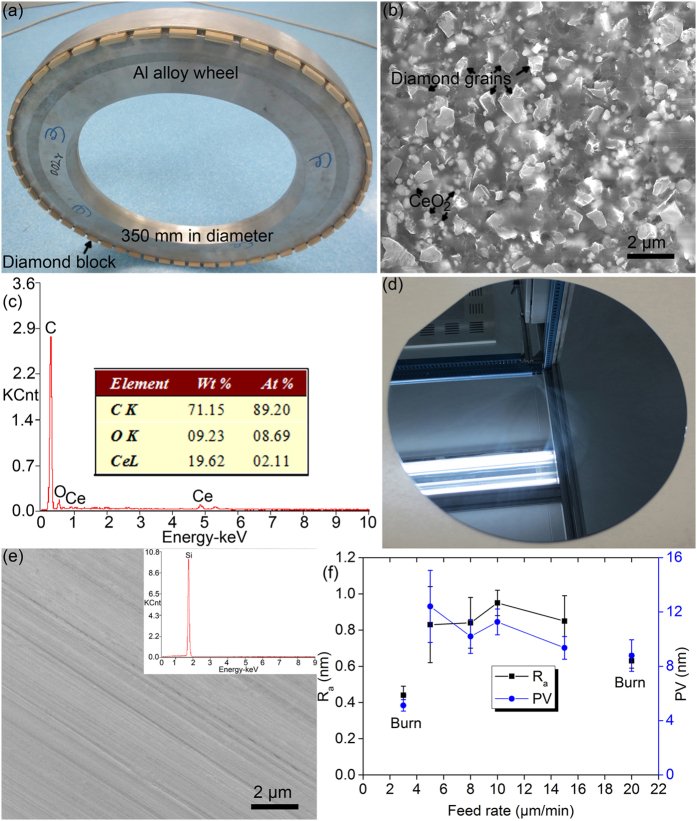
Optical (**a**) and SEM (**b**) images of Sample C2, (**c**) its EDS spectrum in (**b**), optical (**d**) and SEM (**e**) images on a ground Si wafer at a feed rate of 8 μm/min, and (**f**) surface roughness R_a_ and PV as a function of feed rate on the Si wafers ground by Sample C2. The inset in (**e**) showing its corresponding EDS spectrum.

An optical image of a newly developed diamond wheel with ceria is shown in Fig. 2(a), and its SEM image on a diamond block is illustrated in Fig. 2(b). The mesh size of the diamond wheel was 20000, equivalent to an average grain diameter of 760 nm, which is calculated^12^,


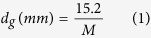


where *d*_*g*_ is the diameter of diamond grains, and *M* is the mesh size of a diamond wheel.

Both the diamond grains and CeO_2_ powders disperse individually well, and agglomeration is absent, indicating a uniform microstructure for the developed diamond wheel. This plays a significant role for the stable and good grinding performance. Weight percentage of ceria is 24.1% in Sample C2, as listed in Table 1, which is consistent with the result of EDS in Fig. 2(c,d) shows a bright surface of a Si wafer ground by Sample C2, like a mirror, revealing an ultrasmooth surface. This is distinct from the dark and burnt surface in Fig. 1(d) ground by Sample S1. Subtle grinding marks are found in Fig. 2(e), indicating the abrasive grinding effect of diamond grains. Cracks are absent on the ground surface, meaning the ductile grinding took place on the ground surface. Oxygen element disappears in the EDS spectrum in the inset of Fig. 2(e), meaning the absence of burn on the ground Si wafer. Surface roughness R_a_ and PV values are pictured in Fig. 2(f). Surface roughness R_a_ and PV fluctuate from 0.83 to 0.95 nm and 9.36 to 12.41 nm, respectively with increasing an infeed rate of wheel from 5 to 15 μm/min, except burn happening at both 3 and 20 μm/min, as shown in Table 2 and Fig. 2(f). The surface roughness R_a_ and PV are stable compared to those in Fig. 1(f), which is in a good agreement with the uniform distribution of diamond grains and ceria particles in Sample C2 (Fig. 2(b)). Moreover, the surface roughness R_a_ is less than 1 nm, resulting in an ultrasmooth surface, which agrees well with the optical image in Fig. 2(d).

**Figure 3:**
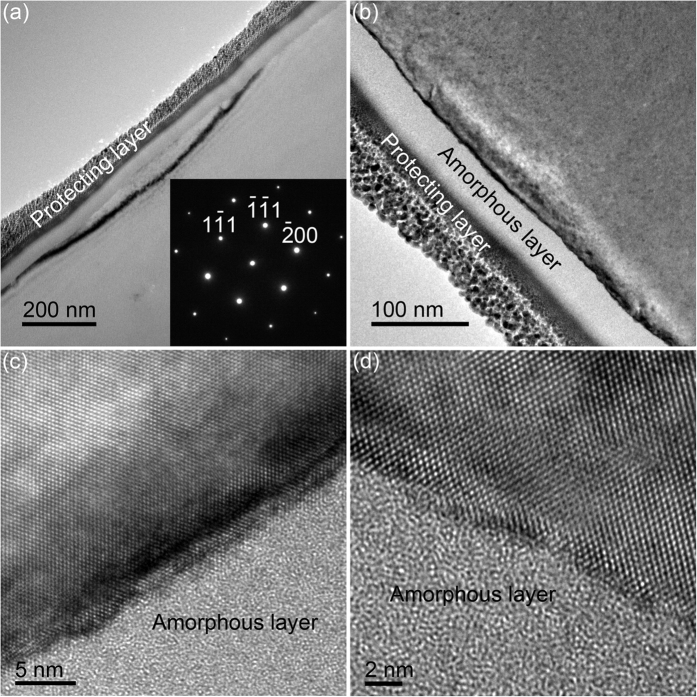
Cross-sectional TEM images at (**a**,**b**) low and (**c,d**) high magnifications formed at an infeed rate of 8 μm/min ground by Sample C2. Inset showing the corresponding SAED pattern of (**a**).

Figure 3 shows the cross-sectional TEM images at low and high magnifications formed at an infeed rate of 8 μm/min ground by Sample C2. There is a uniform amorphous layer at the topmost, as shown in Fig. 3(a). Diamond-cubic Si-I phase is confirmed by selected area electron diffraction (SAED) pattern in the inset of Fig. 3(a), indicating the absence of high pressure phases. This is different from the high pressure phases found in nanoindentation^6^,^7^ and nanoscratch^9^. The thickness of the amorphous layer is 43 nm at the top (Fig. 3(b)), followed by pristine crystalline lattice, as observed in Fig. 3(c,d). This is different from previous reports of ultraprecision grinding^4^,^5^, nanoindentation^6^,^7^,^8^ and nanoscratch^9^, in which there is an amorphous layer at the top and a crystalline damaged layer beneath.

**Figure 4:**
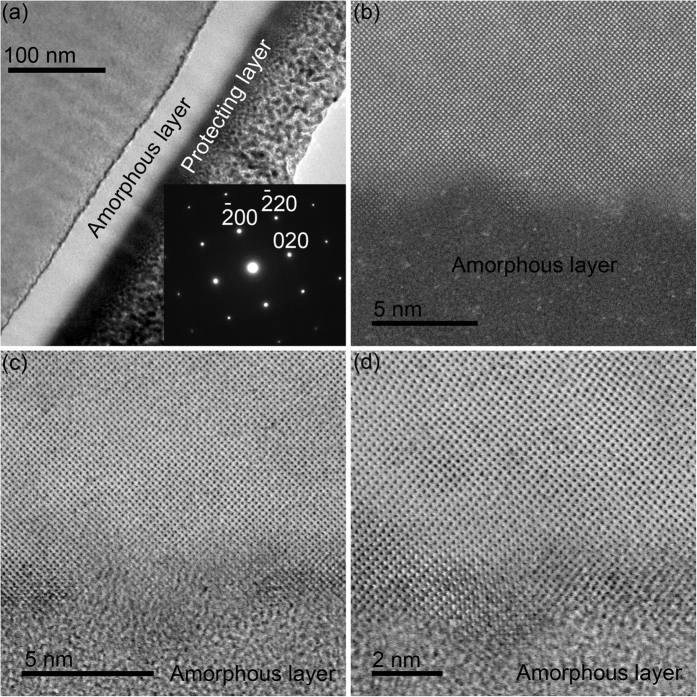
Bright (**a**–**d**) and dark (**b**) fields of cross-sectional TEM images at (**a**) low and (**b–d**) high magnifications induced at an infeed rate of 15 μm/min ground by Sample C2. Inset showing the corresponding SAED pattern of (**a**).

Figure 4 shows the bright and dark fields of cross-sectional TEM images at low and high magnifications induced at an infeed rate of 15 μm/min ground by Sample C2. There is an amorphous layer of 48 nm in thickness at the top (Fig. 4(a)), followed by pristine crystalline lattice underneath, as illustrated in Fig. 4(b–d). Furthermore, diamond-cubic Si-I phase is verified by SAED pattern in the inset of Fig. 4(a). These are in a good agreement with those in Fig. 3.

## Discussion

Elastic modulus of a diamond block is 30.6 GPa, which is measured by the nanomechanical test instrument. This is mainly attributed to the soft nature of phenolic resin bond. For a comparison, the elastic modulus of a Si (100) wafer is 150.2 GPa^13^, which is higher than that of a diamond block. This makes the diamond grains retract during grinding, relieving the aggressive effect of diamond grains on the Si wafers and contributing to forming the nanoscale solely amorphous layer in Si wafers (Figs 3 and 4). A Si wafer is exposed in air, and following equation occurs:


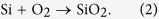


Therefore, silica generates on the surface of Si wafers. Ceria (melting point of 2400 °C) is softer than silica, inflicting less damage on the Si wafers during grinding compared to other harder abrasives, such as La_2_O_3_, Al_2_O_3_, Y_2_O_3_, TiO_2_, etc^14^,^15^. Nevertheless, ceria is soft, whereas realizes high polishing rate, owing to the chemical reaction between ceria and silica, activated under mechanical force and thermodynamics effect during grinding^16^,^17^. Reduction reaction of ceria happens, turning from Ce^4+^ to Ce^3+^ valent states at elevated temperature and mechanical force induced in grinding^18^,^19^:


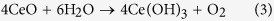



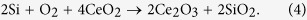


Si^4+^ is easy to eliminate in the form of silicates with deionized water as coolant, as expressed in Eq. (3). For Eq. (4), O bonds in SiO_2_ breaks under mechanical force generated by grinding, accelerating the chemical reaction at the SiO_2_-CeO_2_ interface^16^. This results in the transferring of electrons from the p-orbital of O atoms to the f-orbital of Ce atoms, leading to the reduction reaction of Ce atoms and Si-O-Ce bonding among dangling bonds of O atoms^16^. With the bonding, SiO_2_ lump was pulled out from the SiO_2_ layer on the Si wafers, resulting in a high infeed rate without burn on the surfaces.

The maximum undeformed chip thickness, *h*_*m*_, is used to characterize the grinding conditions and performance^4^,^5^,^20^. The volume fraction of diamond grains in a diamond block, *v* is calculated^4^,^20^,


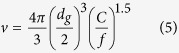


where *C* and f are the amount and fraction of surface active grains per unit area, respectively. With Eq. (5), *C* recasts,


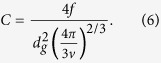


f is used 0.5^4^,^20^. *h*_*m*_ is expressed^4^,^20^,


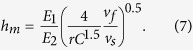


where *r* is a ratio of the width to thickness of an undeformed chip, *E*_*1*_ and *E*_*2*_ are the elastic moduli of a diamond block and a Si wafer, respectively, and *v*_*f*_ and *v*_*s*_ are the infeed rate and speed of the developed diamond wheel, correspondingly. *r* is 1.49^20^. *h*_*m*_ is calculated and listed in Table 1. *h*_*m*_ increases monotonically from 0.63 to 1.1 nm, with increasing an infeed rate from 5 to 15 μm/min. The maximum undeformed chip thickness varies from 0.63 to 1.1 nm, which is at the angstrom level except for the infeed rate at 15 μm/min. This greatly contributes to the formation of nanoscale solely amorphous layer in Si wafers. The maximum undeformed chip thickness is 0.8 nm at an infeed rate of 8 μm/min, which is lower than 1.1 nm at an infeed rate of 15 μm/min, resulting in the lower thickness (43 nm) of solely amorphous layer of the former than the latter (48 nm), as experimentally demonstrated in Figs 3 and 4, respectively. The undeformed chip thickness is 0.49 nm at an infeed rate of 3 μm, which is the lowest in all undeformed chip thicknesses. The undeformed chip thickness is lower, the grinding energy is higher 4, 5. When the undeformed chip thickness is less than a critical value, the grinding energy would increase sharply, resulting in the burn happening. On the other hand, burn occurs when the grinding removal rate is lower than an infeed rate, in terms of rubbing happening rather than grinding. For instance, the infeed rate of 20 μm/min might exceed the grinding removal rate, leading to the burn taking place.

In summary, two novel phenolic resin bond diamond wheels are developed, and ultraprecision grinding is performed on Si wafers using the developed wheels. Nanoscale solely amorphous layer is obtained in Si wafers by Sample C2, followed by pristine crystalline lattice, which is characterized by HRTEM. Burn happens on all the ground Si wafers by Sample S1. For a comparison, surface roughness R_a_ is less than 1 nm induced by Sample C2, and the surface looks like a mirror, forming bright and ultrasmooth surfaces on Si wafers. It is therefore, ceria plays a significant role for achieving bright surface on Si wafers. The newly developed diamond wheel with ceria is beneficial for semiconductor and microelectronics industries.

## Methods summary

As-received Si (100) wafers were used as specimens for ultraprecision grinding. The surface roughness R_a_ and peak-to-valley (PV) values of Si wafers were 0.38 ± 0.05 and 3.9 ± 0.4 nm, respectively. Phenolic resin was employed for the bond. Diamond grains, phenolic resin and CeO_2_ were mixed uniformly and pressed into diamond blocks, with dimensions of 18 × 8 × 3 mm^3^ at room temperature. All the diamond blocks were thermally solidified at temperature varying from 220 to 240 °C for 2 h. After solidification, the concentration percentage of diamond grains was 150 in each diamond block, equivalent to a volume fraction of 37.5%. Forty eight blocks were glued and distributed uniformly along a notch at the periphery of an aluminum wheel with a diameter of 350 mm, forming two developed diamond wheels with ceria and silica, respectively. The notch was 3.5 mm in width and 3 mm in depth, resulting in 5 mm in height of a diamond block exposed outside. The diamond wheel was mounted on an ultraprecision grinder (Okamoto, VG401 MKII, Japan) with face runout of 50 nm via vacuum chuck. Prior to grinding, the diamond wheel was trued by an iron cast plate using silicon carbide (SiC) slurry. The wheel and table speeds were 40.3 m/s and 100 rpm, respectively listed in Table 2, and deionized water was applied as coolant during ultraprecision grinding. Four Si wafers were repeatedly ground at a fixed infeed rate to verify the grinding performance. Surface roughness of Si wafers was measured by non-contact surface profilometry (Zygo, NewView5022, USA) prior to and after grinding. Surface microstructure was characterized by field emission environmental scanning electron microscopy (FEI, Quanta 200 FEG, Netherlands) equipped with EDS spectrum. Cross-sections of the ground Si wafers were characterized by both high resolution TEM (FEI, Tecnai F20, Netherlands) and ultrahigh resolution TEM (JEOL, JEM-ARM200F, Japan). TEM specimens were prepared using focused ion beam (FIB, FEI, Helios 600i, Netherlands) technique, and then thinned by a Gatan Model 691 precision ion polishing system. Elastic modulus of a diamond block was measured by a nanomechanical test instrument (TI 950, TriboIndenter, Hysitron, Minneapolis). Loading, dwelling and unloading time was 5, 2, 5 s, respectively during nanoindentation at a peak load of 3 N.

## Additional Information

**How to cite this article**: Zhang, Z. *et al*. Nanoscale solely amorphous layer in silicon wafers induced by a newly developed diamond wheel. *Sci. Rep.* **6**, 35269; doi: 10.1038/srep35269 (2016).

## Figures and Tables

**Table 1:** Specifications of diamond wheels and grinding conditions.

Sample	Additive (wt.%)	Additive (vol.%)	Resin bond (vol.%)	Feed rate (μm/min)
S1	SiO_2_ 8.2	SiO_2_ 13	49.5	3, 5, 8, 10
C2	CeO_2_ 24.1	CeO_2_ 12	50.5	3, 5, 8, 10, 15, 20

**Table 2:** Grinding conditions, surface roughness and calculated undeformed chip thickness induced by Sample C2.

Wheel speed (m/s)	Table speed (rpm)	infeed rate of wheel (μm/min)	Surface roughness (nm)		Calculated undeformed chip thickness (nm)
			R_a_	PV	
40.3	100	3	0.44 ± 0.05	5.12 ± 0.42	0.49
		5	0.83 ± 0.21	12.41 ± 2.65	0.63
		8	0.84 ± 0.14	10.2 ± 1.25	0.8
		10	0.95 ± 0.07	11.27 ± 0.95	0.89
		15	0.85 ± 0.14	9.36 ± 0.84	1.1
		20	0.63 ± 0.04	8.79 ± 1.16	1.27
